# Wavelength and pulse duration tunable ultrafast fiber laser mode-locked with carbon nanotubes

**DOI:** 10.1038/s41598-018-21108-3

**Published:** 2018-02-09

**Authors:** Diao Li, Henri Jussila, Yadong Wang, Guohua Hu, Tom Albrow-Owen, Richard C. T. Howe, Zhaoyu Ren, Jintao Bai, Tawfique Hasan, Zhipei Sun

**Affiliations:** 1 State Key Lab Incubation Base of Photoelectric Technology and Functional Materials, and Institute of Photonics and Photon-Technology, Northwest University, 710069 Xi’an, China; 2 Department of Electronics and Nanoengineering, Aalto University, Tietotie 3, FI-02150 Espoo, Finland; 3 MOE Key Laboratory of Material Physics and Chemistry under Extraordinary Conditions, and Shaanxi Key Laboratory of Optical Information Technology, School of Science, Northwestern Polytechnical University, 710072 Xi’an, China; 4 Cambridge Graphene Centre, University of Cambridge, 9 JJ Thomson Avenue, CB3 0FA Cambridge, UK; 5 QTF Centre of Excellence, Department of Applied Physics, Aalto University, FI-00076 Aalto, Finland

## Abstract

Ultrafast lasers with tunable parameters in wavelength and time domains are the choice of light source for various applications such as spectroscopy and communication. Here, we report a wavelength and pulse-duration tunable mode-locked Erbium doped fiber laser with single wall carbon nanotube-based saturable absorber. An intra-cavity tunable filter is employed to continuously tune the output wavelength for 34 nm (from 1525 nm to 1559 nm) and pulse duration from 545 fs to 6.1 ps, respectively. Our results provide a novel light source for various applications requiring variable wavelength or pulse duration.

## Introduction

Ultrafast fiber lasers play key roles not only in scientific research, but also in commercial applications, including in materials processing^1^, spectroscopy^2^, sensing^3^, optical signal processing^4^ and optical fiber communication systems^5^. The generation of ultrafast pulses in fiber lasers is usually dependent on passive mode-locking technology, where a nonlinear saturable absorber (SA) serves as a passive optical modulation device to turn continuous wave (CW) output into a periodic pulse train. Currently, the most commonly used SAs are semiconductor saturable absorber mirrors (SESAMs)^1,6–8^. However, SESAMs suffer from drawbacks in complex and expensive fabrication technology (e.g., molecular beam epitaxial growth^9^) and narrow operation bandwidth (a few tens of nanometers^10^). In the past two decades, carbon-based nanomaterials such as single wall carbon nanotubes (SWNTs)^11–29^ and graphene^25,29–43^ have exhibited their merits as promising candidates for SAs. For instance, SWNTs present a broad operation bandwidth^44–46^ by controlling the diameter distribution. In comparison with conventional SAs (e.g., SESAM), SWNTs exhibit significant advances for SA technologies in terms of fast recovery time (down to a few hundred femtoseconds)^47,48^, low non-saturable loss^28^, easy and cost-effective fabrication and integration^28,29,49,50^.

For various applications, novel ultrafast lasers with flexible output performance are more desirable^51,52^. To demonstrate wavelength-tunable pulsed lasers, several methods have been investigated. For example, Refs^53–55^ demonstrated wavelength-tunable ultrafast lasers mode-locked by nonlinear polarization rotation (NPR), in which the intrinsic spectral filter induced by the intracavity fiber birefringence was adopted to tune the wavelength. Ref.^56^ reported a fiber Bragg grating-based filter for wavelength and duration tunable ultrafast pulse generation, where a carbon nanotube SA was used as the mode-locker. Nevertheless, these nonlinearity and birefringence dependent wavelength selection technologies encountered performance variation to temperature and environmental fluctuations^57^. In addition, the output wavelength could not be precisely tuned by controlling the polarization state (i.e., birefringence). Moreover, the relatively narrow reflection bandwidth of fiber Bragg grating limits the pulse duration to picosecond range.

In order to overcome these issues, a promising solution is to combine a highly precise tunable filter with a broadband mode-locker that can support a continuous wavelength selection over a wide span for ultrafast pulse generation. At 1.55 μm telecommunication band, the broad gain spectrum of Erbium doped fiber (EDF) enables ultrafast lasers to be mode-locked over a wide wavelength range^45^. Particularly, SWNTs with a diameter range of 1.0–1.3 nm exhibit a peak absorption at ~1567 nm^58^, which can optimally match the spectral range of EDF lasers (1530–1565 nm) and act as an ideal candidate SA for tunable ultrafast laser application.

In this paper, we use a wideband tunable filter and a broadband SWNT-SA for ultrafast EDF laser at the 1.55 μm telecommunication window, in which both the output wavelength and pulse duration are flexibly tunable. We achieve a continuous wavelength tuning over a 34 nm bandwidth from 1525 nm to 1559 nm, which is only limited by the filter. The pulse duration is also adjustable by controlling the variation of spectral bandwidth via the tunable filter. Ultrafast pulses with duration between 545 fs and 6.1 ps are obtained with a corresponding output spectral bandwidth variation from 6 nm to 1.2 nm. Our laser configuration provides an advanced platform for the development of practical light sources with controllable performance in wavelength and time domains.

## Results and Discussion

### SWNT polymer composite SA

**Figure 1:**
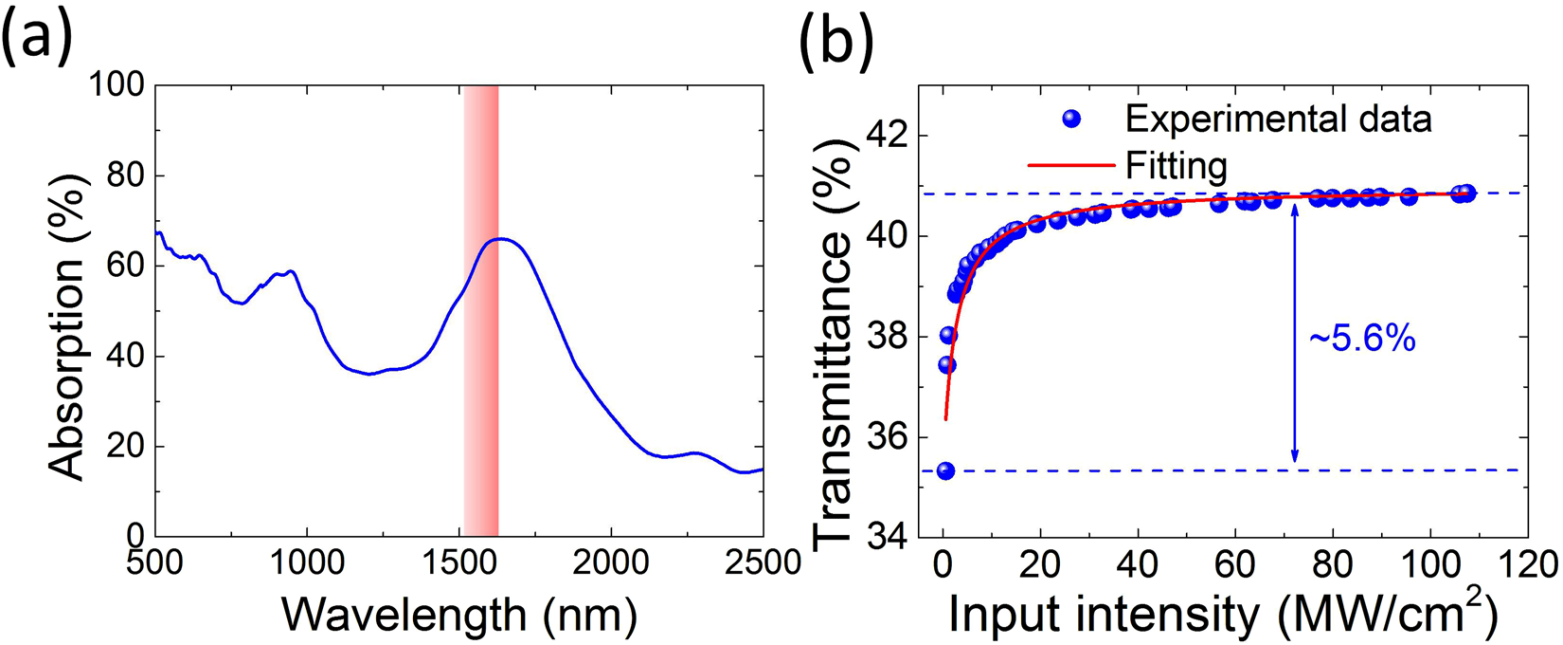
(**a**) Optical absorption spectrum of our SWNT-CMC polymer composite film (pink rectangular area indicates our operation laser wavelength range). (**b**) Transmittance of the film as a function of the input intensity.

The SWNT-based SA preparation is similar to the processes reported in Refs^29,59^. We disperse ~0.03 wt.% laser ablation SWNTs (~1.3 nm mean diameter)^60^ in deionized water with ~0.7 wt.% sodium-carboxymethycellulose (Na-CMC) polymer. In comparison with the commonly used host polymer of polyvinyl alcohol (PVA), Na-CMC can function as both dispersant and host polymer for free-standing SWNT-SA fabrication^61^. Further, Na-CMC is more stable and has higher transmittance at 1.5 μm than PVA as PVA has strong moisture absorption^29^. The choice of SWNTs ensures a broad absorption peak near 1.55 μm^62,63^, measured with UV-Vis spectrophotometer (Fig. 1a). The sonication is carried out for one hour using a tip sonicator (Branson 450A, 20 kHz) with ~50 W power at room temperature. The as prepared dispersion does not exhibit any visible SWNT aggregates. This is then centrifuged in a swing bucket rotor at 30 k rpm using an Optima-Max-E ultracentrifuge (Beckman Coulter) to filter out the residual bundles and impurities. The top ~70% of the dispersion (~6 ml) is decanted for composite fabrication. Slow evaporation of water under ambient temperature for one week produces a free-standing SWNT-CMC SA.

Nonlinear absorption of the fabricated SWNTs-CMC SA is measured by using a home made amplified ultrafast fiber laser with a balanced twin detector setup. The maximum laser output power is ~15 mW with 530 fs pulse duration and 62 MHz repetition rate at a central wavelength of 1550 nm. Optical transmittance as a function of the input intensity is plotted in Fig. 1b. The experimental data is fitted with the equation given in Ref.^45^. The result shows a modulation depth of ~5.6%, a saturation intensity of 1.2 MW/cm^2^ and non-saturable loss of ~59%. Note that it is possible to further reduce the non-saturable loss while maintaining the modulation depth through optimization of the SWNT device fabrication (*e.g.*, nanotube diameter control, reduced scattering and coupling losses^27,29^).

### Tunable fiber laser setup

**Figure 2:**
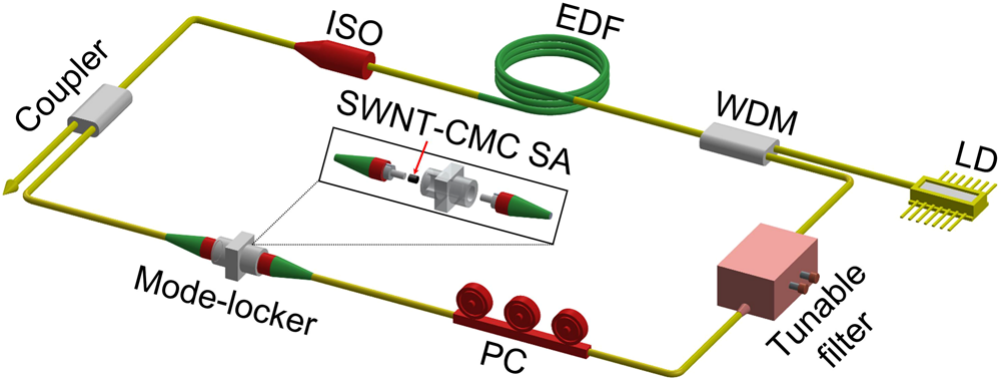
The schematic setup of the mode-locked fiber laser. LD, laser diode; WDM, wavelength division multiplexer; EDF, Erbium doped fiber; ISO, polarization independent isolator; PC, polarization controller.

**Figure 3:**
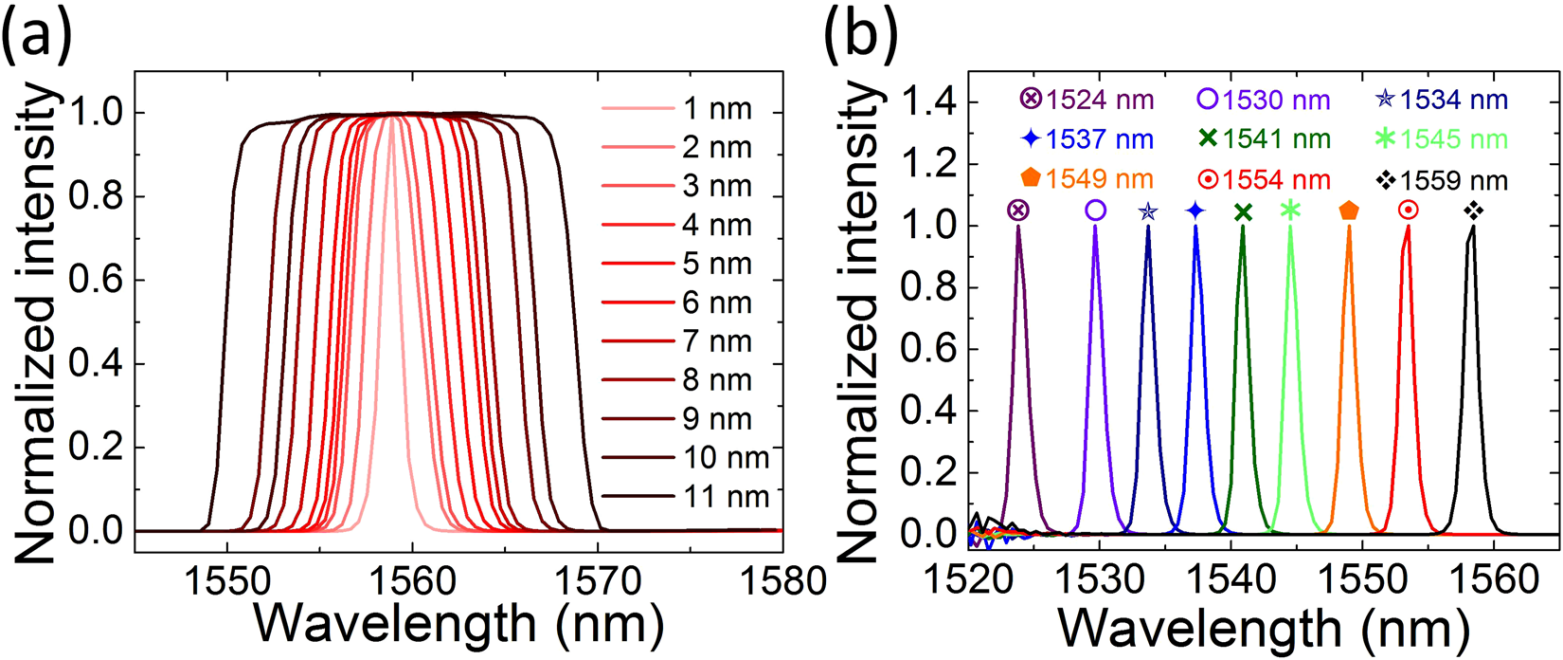
Typical transmission spectra of the TBWF. (**a**) Bandwidth tunable spectra with fixed central wavelength of 1559 nm. FWHMs of the transmission spectra are continuously enlarged from 1 nm to 11 nm. (**b**) The central wavelength of the transmission spectra is tuned from 1524 nm to 1559 nm as the spectral bandwidth is compressed to ~1.2 nm.

 A schematic of the laser setup is shown in Fig. 2. A segment of ~0.8 m EDF is used as the gain medium, which is pumped through a 980/1550 nm fused fiber wavelength division multiplexer (WDM) by a 980 nm laser diode (LD). A polarization independent isolator (ISO) is connected subsequently to ensure unidirectional propagation of the light in the circular cavity. A polarization controller (PC) is used to optimize the laser mode-locking operation by adjusting the state of polarization. The SWNT-CMC polymer composite film is sandwiched between two fiber connectors to form an all-fiber integrated SA device. The intracavity light is extracted via the 10% port of a 10/90 fiber coupler. To investigate the wavelength and pulse duration tunability in the mode-locking scheme, a tunable bandpass and wavelength filter (TBWF, PriTel) is employed to selectively control the output pulse performance in wavelength and time domains. Tuning the tilt angle of the filter by rotating two adjustable micrometer screws controls the central wavelength and the spectral bandwidth. The bandwidth of the transmission spectrum can be varied with symmetric enlargement and compression of the filter passband. Figure 3(a) shows the typical transmission spectra with different FWHMs from 1 nm to 11 nm with the central wavelengths fixed at 1559 nm (the maximum wavelength available with our filter). Note that similar bandwidth variable transmission spectra are also available if the central wavelength is tuned to smaller values but larger than 1541.5 nm. Wavelength tuning of the transmission spectrum with fixed profile is a result of the filter passband edges moving along the same direction. The maximum tunable range is acquired for 35 nm (1524–1559 nm) when the bandwidth is compressed to ~1.2 nm, as shown in Fig. 3(b). These experimental results demonstrate the TBWF is an effective tool for independent bandwidth and wavelength tuning.

The total laser cavity length is ~11.8 m. Besides the EDF with group velocity dispersion (GVD) of ~53.6 ps^2^/km, all the other fibers in the cavity are standard telecommunication single mode fiber (SMF-28e) with GVD of ~−23 ps^2^/km. The net cavity GVD is calculated to be ~−2.1 × 10^−1^ ps^2^. To characterize the laser performance, a high resolution (0.03 nm) optical spectrum analyzer (Anritsu, MS9740A) and a second-harmonic generation autocorrelator (APE, Pulse-check 50) are utilized to measure the output spectrum and pulse duration. An oscilloscope and a frequency spectrum analyzer (Anritsu, MS2692A) connected with an ultrafast (>25 GHz) photodetector are employed to record the output pulse train and radio frequency spectra. A power meter (Ophir, Nova II) is used to measure the output power.

### SWNT-CMC SA mode-locked fiber laser

**Figure 4:**
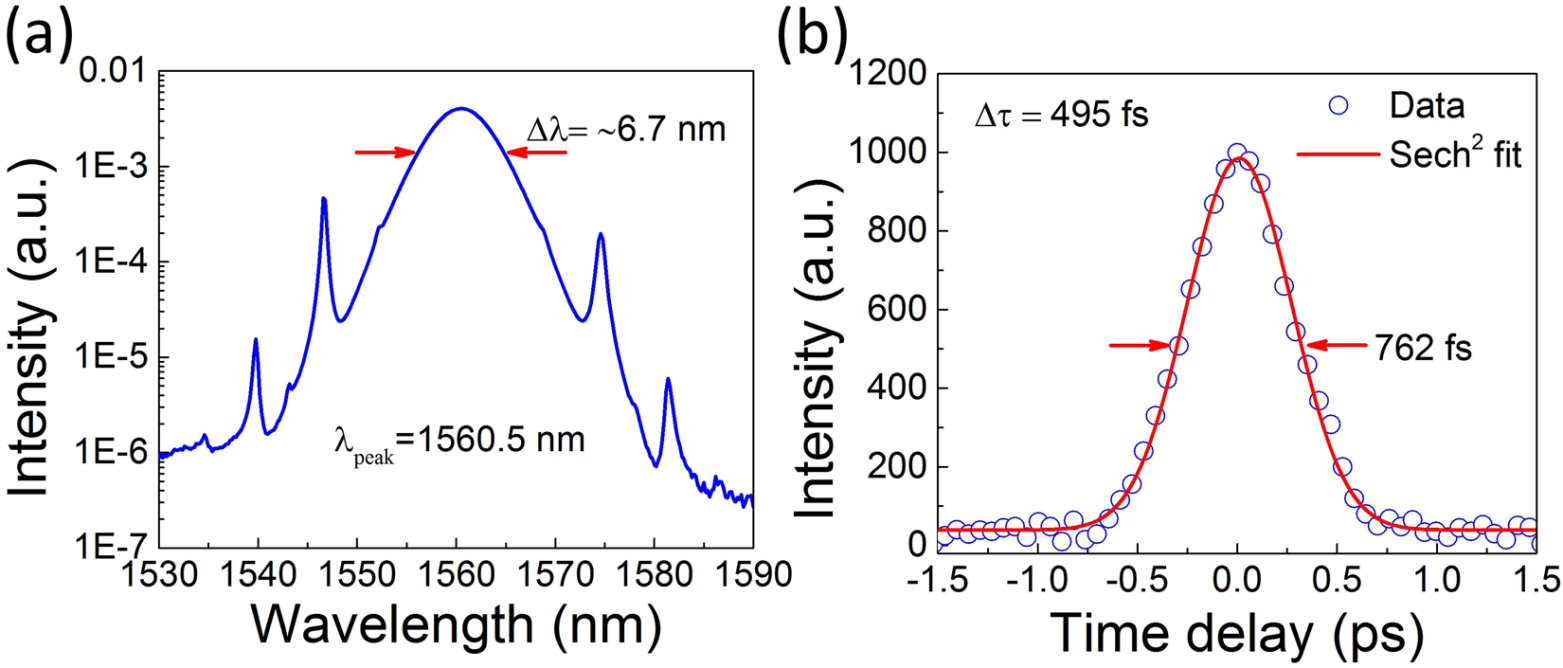
(**a**) Output spectrum and (**b**) pulse duration measurements of the mode-locked laser without TBWF.

Mode-locked laser performance of the cavity without the TBWF is first characterized. CW laser output is obtained when the pump power reaches ~5.3 mW. When the pump power is further increased to ~17.1 mW, the CW light is converted to a mode-locked pulse train with an output power of ~1.26 mW. To stabilize the pulse train, intracavity birefringence needs to be modified by adjusting the intracavity PC. After, stable mode-locking is well maintained without adjustment of PC, even when we further increase the pump power or tune the filter. Stable CW mode-locking operation is observed in a pump power range of 17.1–56.9 mW. Figure 4 shows the output laser performance measured at the pump power of 33 mW. The laser mode-locks at the central wavelength of 1560.5 nm, with a full width at half maximum (FWHM) of ~6.7 nm; Fig. 4(a). The sidebands (located at 1539.78 nm, 1546.58 nm, 1574.58 nm and 1581.38 nm) confirm a soliton-like mode-locking, revealing a typical character of anomalous dispersion cavities^64^. Figure 4(b) depicts the measurement results of the pulse duration, which can be well-fitted to a sech^2^ temporal profile. The autocorrelation trace implies an FWHM pulse duration of 495 fs. The time bandwidth product (TBP) of the soliton-like pulse is 0.408, slightly larger than the sech^2^ transform-limited TBP value of 0.315. This indicates that the output pulses are slightly chirped.

### Wavelength tunable performance of the mode-locked fiber laser with the filter

**Figure 5:**
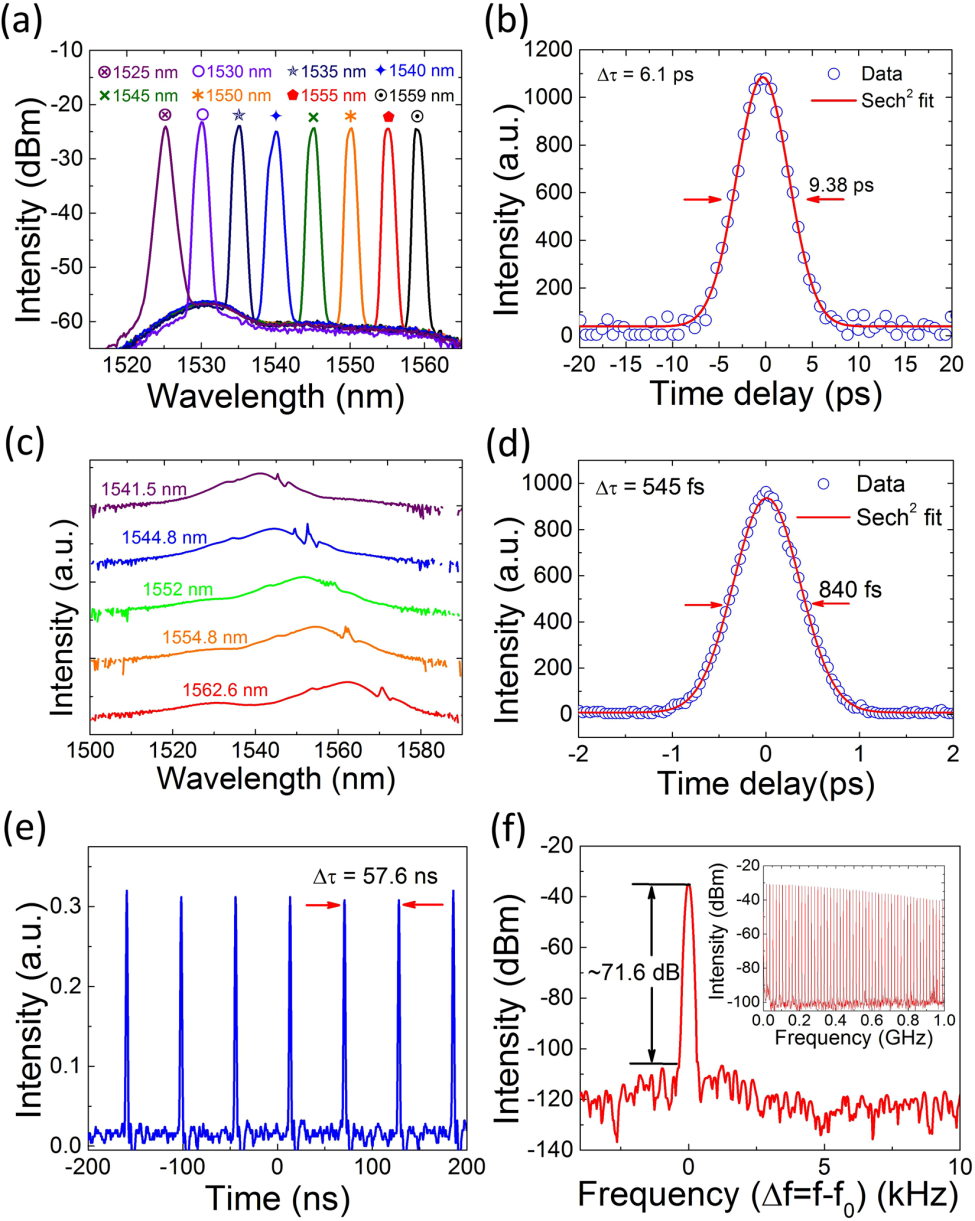
Wavelength tunability of the mode-locked fiber laser. (**a**) Mode-locked laser output spectra for a 34 nm span, FWHMs of the output spectra are ~1.2 nm. (**b**) Second harmonic autocorrelation trace of mode-locked pulse at 1545 nm wavelength. (**c**) Output spectra over a 21.1 nm span. FWHMs of the output spectra are 6 nm. (**d**) Second harmonic autocorrelation trace of mode-locked pulse at 1544.8 nm. (**e**) Output pulse train (corresponding to a repetition rate of 17.4 MHz). (**f**) Radio frequency spectrum at the fundamental repetition rate of f_0_ (f_0_ = 17.4 MHz) with 100 Hz resolution bandwidth. Inset: broadband frequency spectrum (up to 1 GHz) with 100 kHz resolution bandwidth.

The pump power range associated with stable mode-locking operation increases when the filter is inserted in the cavity. However, to compare the laser performance, we measure the wavelength tunability results (Fig. 5) at a pump power of 33 mW. Output wavelength of the mode-locked laser is tuned for a 34 nm span, covering the range from 1525 nm to 1559 nm as shown in Fig. 5(a). FWHMs of all the output spectra are ~1.2 nm, which are approximately half of the filter bandwidth. This is consistent with the previous ultrafast fiber laser demonstration with an intracavity filter^65^. These broad wavelength tunable results confirm the wide operation bandwidth of the demonstrated SWNT-based SA. Noting that the experimentally acquired tuning range is limited by the passband width of the TBWF (see Fig. 3(b)). Therefore, a tunable wavelength span larger than 34 nm is also possible. Figure 5(b) plots a representative pulse duration measurement, the sech^2^ fitting result gives a pulse duration of 6.1 ps, which is the longest pulse generated in this laser (corresponding to the narrowest spectral bandwidth of 1.2 nm). In further investigation, we intentionally enlarge the spectral bandwidth in the available range and then investigate the tunability of central wavelength at fixed bandwidth. The results demonstrate that the spectral bandwidth can be continuously changed between 1.2 and 6 nm (limited by the filter bandwidth tuning range of ~2 nm and 11 nm). Figure 5(c) shows the experimentally measured spectra of the continuously tuned laser from 1541.5 nm to 1562.6 nm with a maximum bandwidth of 6 nm. The measured autocorrelation trace in Fig. 5(d) gives a pulse duration of 545 fs, which is longer than the pulse (495 fs) without filtering. This expected pulse duration extension is attributed to the spectral filtering and anomalous dispersion increase caused by the TBWF and its fiber pigtail.

Figure 5(e) presents a typical mode-locked pulse train. The 57.6 ns pulse interval (corresponding to a repetition rate of 17.4 MHz) is consistent with the optical cavity round trip time. To characterize the stability of the mode-locking, we measure the radio frequency spectrum at the fundamental frequency of f_0_ with a resolution bandwidth of 100 Hz (shown in Fig. 5(f)). As can be seen, the signal-background noise ratio (SNR) of the output pulses is ~71.6 dB (with a contrast of >10^7^), indicating highly stable mode-locking. We then measure the harmonic frequencies with a span of up to 1 GHz at 100 kHz resolution bandwidth (see the inset of Fig. 5(f)), which confirms a stable CW mode-locking of the laser. During our experiment, no performance degradation is observed, indicating the good stability of our SWNT-based SA.

### Pulse-duration tunable performance of the mode-locked laser

**Figure 6:**
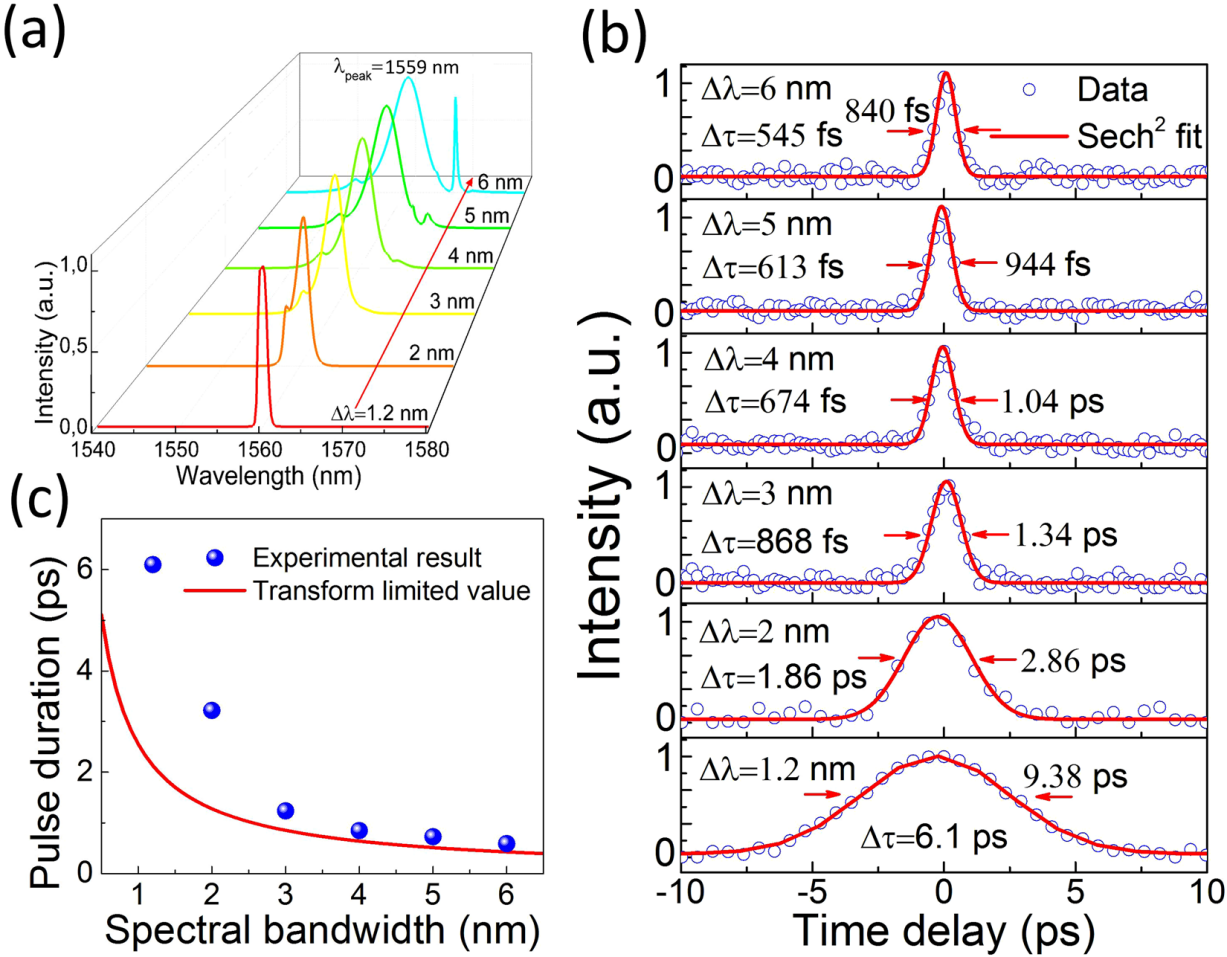
Pulse-duration tunable mode-locked output performance. (**a**) FWHM bandwidth tunable from minimum 1.2 nm to maximum 6 nm with a symmetric distribution at central wavelength of 1559 nm. (**b**) Pulse duration measurement corresponding to different spectral bandwidths. (**c**) Output pulse duration as a function of spectral FWHM. Blue spheres show the experimental value, red curve indicates the transform limited pulse durations.

The spectral bandwidth can be readily tuned by controlling the passband of the TBWF. Our experimental observation shows that the FWHM of the mode-locked output spectral bandwidth is continuously tunable from the maximum value of 6 nm to the minimum of 1.2 nm. The slight reduction of the maximum bandwidth (from 6.7 nm to 6 nm) is caused by the unavoidable spectral filtering from the TBWF. Upon further tuning the spectral bandwidth to <1.2 nm, the mode-locking pulse train becomes unstable and finally collapses. This is due to the rise of intracavity loss from a strong wave filtering^66^ as the passband of the filter is compressed. Figure 6(a) performs the mode-locked output spectra with bandwidths of 1.2, 2, 3, 4, 5, and 6 nm centered at the same wavelength of 1559 nm. It is noteworthy to mention that such large spectral bandwidth tuning (from 1.2 nm to 6 nm) at peak wavelengths shorter than 1541.5 nm (*e.g.*, 1530 nm) is not achieved in our experiment, possibly due to the gain filtering effect. To investigate the relationship between the output spectral bandwidth and the pulse duration, we measure the autocorrelation traces of the mode-locked pulses at different bandwidths obtained in Fig. 6(a). The corresponded results are plotted in Fig. 6(b). The pulse durations as a function of the respective spectral bandwidth are then analytically shown in Fig. 6(c) (blue spheres). We also theoretically calculate the pulse duration Δτ with the following formula ${\rm{\Delta }}{\rm{\tau }}=\tfrac{{\lambda }^{2}}{c\cdot {\rm{\Delta }}\lambda }\times 0.315$, where Δτ is the pulse duration, *λ* the central wavelength (i.e., 1559 nm), *c* the velocity of light in vacuum and Δ*λ* the FWHM of the output spectrum. It can be seen that the experimental pulse duration results corresponding to the 3 nm, 4 nm, 5 nm and 6 nm spectral bandwidth are quite close to the transform limited values at the sub-picosecond range (shown in red, Fig. 6(c)). However, when the output spectral bandwidth is further reduced to 2 nm and 1.2 nm, the experimental pulse durations are longer than that of the transform limited values. This TBP fluctuation is caused by the variations of a few factors including the filter bandwidth and the optical and cavity dispersion^56^. The flexible and large range wavelength and pulse duration tunability in our experiment provides a new approach for developing novel ultrafast source. SWNT polymer composite SA and in-line tunable filter play the key roles for tunable pulse generation in a compact and environmentally insensitive fiber cavity. Note that the wavelength and pulse duration tunable fiber laser design is also applicable to other nanomaterial^67^ optical switch based fiber lasers (e.g., graphene^29,33–43,68^, black phosphorus^69–72^, MoS_2_^36,73–76^, WS_2_^74,77^, MoSe_2_^74,78^, WSe_2_^79^, and their heterostructures^35,80^).

## Conclusion

We report a wavelength and pulse duration tunable fiber laser passively mode-locked by SWNT-based SA. The output wavelength of the pulses can be continuously tuned over a 34 nm bandwidth (from 1525 nm to 1559 nm). We observe a wide pulse duration variation between 545 fs and 6.1 ps with a corresponding spectral bandwidth tunable from 6 nm to 1.2 nm. The wavelength and pulse duration tunable fiber laser demonstrated in this work can be used in basic research as well as commercial applications, such as spectroscopy, optical singal processing and optical fiber communication systems.

### Data availability

The datasets generated during the current study are available from the corresponding author on reasonable request.
